# Measurements of intrahost viral diversity require an unbiased diversity metric

**DOI:** 10.1093/ve/vey041

**Published:** 2019-01-30

**Authors:** Lei Zhao, Christopher J R Illingworth

**Affiliations:** 1 Department of Genetics, University of Cambridge, Downing Street, Cambridge, UK; 2 Department of Applied Mathematics and Theoretical Physics, University of Cambridge, Wilberforce Road, Cambridge, UK

**Keywords:** virus diversity, sequence data, polymorphism, entropy

## Abstract

Viruses exist within hosts at large population sizes and are subject to high rates of mutation. As such, viral populations exhibit considerable sequence diversity. A variety of summary statistics have been developed which describe, in a single number, the extent of diversity in a viral population; such measurements allow the diversities of different populations to be compared, and the effect of evolutionary forces on a population to be assessed. Here we highlight statistical artefacts underlying some common measures of sequence diversity, whereby variation in the depth of genome sequencing may substantially affect the extent of diversity measured in a viral population, making comparisons of population diversity invalid. Specifically, naive estimation of sequence entropy provides a systematically biased metric, a lower read depth being expected to produce a lower estimate of diversity. The number of polymorphic loci per kilobase of genome is more unpredictably affected by read depth, giving potentially flawed results at lower sequencing depths. We show that the nucleotide diversity statistic *π* provides an unbiased estimate of diversity in the sense that the expected value of the statistic is equal to the correct value of the property being measured. Our results are of importance for studies interpreting genome sequence data; we describe how diversity may be assessed in viral populations in a fair and unbiased manner.

## 1. Introduction

Many viruses form large within-host populations and evolve under the influence of high mutation rates. As a consequence, within-host viral populations may contain a large amount of sequence diversity (Lauring, Frydman, and Andino 2013). Sequence diversity has a close relationship with the evolution of viral populations; changes in host-mediated pressure on the virus may cause changes in sequence diversity (Poirier and Vignuzzi 2017), while diversity itself may enable more rapid adaptation to new selective pressures (Illingworth 2015). The extent of within-host diversity has been explored in a range of viral diseases (Shankarappa et al. 1999; Bull et al. 2012; McWilliam Leitch and McLauchlan 2013; Grad et al. 2014; Pennings, Kryazhimskiy, and Wakeley 2014; Raghwani et al. 2016; Debbink et al. 2017).

While sequence diversity is complex property, there exist a range of statistical measures of diversity, each capturing the diversity of a population in a single numerical value. Such measures, which include the number of polymorphisms per thousand bases, sequence entropy, and the population genetics parameter *π*, allow for the simple evaluation of changes in population diversity. For example, the amount of diversity in one population may be compared to the amount of diversity in another. In an evolving population, increases and decreases in diversity may be measured over time (Gall et al. 2013; Maldarelli et al. 2013).

Measuring sequence diversity requires an accurate representation of the population under study, acquired through genome sequencing. A broad range of publications have acknowledged, measured, or sought to correct noise in genome sequence data (Beerenwinkel and Zagordi 2011; Archer et al. 2012; Illingworth et al. 2017; Zanini et al. 2017). Accurate experimental techniques have been highlighted as a necessary first step to measuring viral sequence diversity (McCrone and Lauring 2016).

Here we show that an accurate experimental protocol for sequencing is not sufficient to obtain a correct assessment of viral diversity; in addition, an unbiased diversity metric needs to be applied. While previous studies have highlighted biases in naive estimators of entropy statistics (Miller 1955; Harris 1977; Grassberger 1988; Herzel, Schmitt, and Ebeling 1994; Nemenman et al. 2008), their importance in the analysis of viral sequence data has not been fully investigated, and the use of raw entropy statistics is common in the virological literature. Data may be used to evaluate the within- or between-host diversity of populations (Renzette et al. 2017). We here consider three measures of sequence diversity, demonstrating that potentially serious bias may arise from realistic depths of genome sequencing. We highlight the need to account for the stochastic nature of diversity statistics and outline steps via which the diversity of one population may be accurately compared to that of another.

## 2. Methods

### 2.1 Sequence data

Viral sequence data were downloaded from publicly available datasets. The HIV data analysed was that collected after 2639 days in Patient 1 of the dataset described by Zanini et al. (2015); pre-calculated variant frequencies were used for this analysis. The influenza data analysed was from the sample MH5817_20140113_A (SRR6121395) of the dataset described by McCrone et al. (2018). Short-read data from this dataset were aligned using the BWA algorithm (Li and Durbin 2009), with variant frequencies being calculated using the SAMFIRE software package (Illingworth 2016).

### 2.2 Downsampling of data

Downsampling of data was conducted by a simple multinomial process. Supposing the read depth at the locus *l* to be *N_l_*, and that sequence data reported $n_{l,i}$ copies of each of the alleles *i* in the set {A,C,G,T} at position *l*, we calculated the observed allele-based probabilities ${\hat{p}}_{l,i}=n_{l,i}/N_{l}$. A downsampled set of data was generated by choosing a depth *N_d_* for downsampling. At each locus for which *N_l_ *>* N_d_*, a random multinomial draw of depth *N_d_* and with probabilities $p_{l,i}$ was made in order to sample allele frequencies. For each locus for which $N_{l}\leq N_{d}$, the original sequence data were retained. Downsampling was conducted to depths for which at least 90% of loci in the genome had *N_l_ *>* N_d_*.

## 3. Results

Assuming sequencing to have been conducted in an error-free manner, we evaluated the robustness of three statistics: sequence entropy, the number of polymorphic loci per kb, and the nucleotide diversity statistic *π*. In our calculations, we use *L* to denote the length of a hypothetical viral genome. At a given locus, we suppose the underlying frequencies of each nucleotide *i* to be given by *p_i_*. Given sequencing of depth *N*, we suppose that *n_i_* copies of each nucleotide have been observed.

### 3.1 Shannon entropy

The Shannon entropy of a population is derived from information theory, and assesses the level of ‘disorder’ in a population (Shannon 1948); this measure has been used to assess changes in viral sequence diversity over time (Gall et al. 2013). At the locus *l*, the entropy may be calculated as

(1) $$H_{l}=-\sum_{i=1}^{4}p_{i}\;\text{log}(p_{i})$$

where the sum is calculated across the frequencies of the four possible nucleotides. Supposing that full haplotype information for the virus is not available, a genome-wide measure of entropy may then be calculated, computing the mean of this statistic across all sites (McCrone and Lauring 2016).

(2) $$H=\sum_{l=1}^{L}H_{l}/L$$

where genome sequencing is applied to a population, the resulting observations are stochastic in nature, arising from a random sampling process; if sequencing is error-free this can be represented as a multinomial sample collected from the viral population. The value of the entropy calculated from the sequence data, which we denote $\hat{H}$, is therefore a random variable, which may by chance be higher or lower than the true sequence entropy.

Calculations show that if a naive estimator is used, the expected value of this ‘measured’ sequence entropy, or $E(\hat{H_{l}})$, falls between two limits, such that

(3) $$\begin{array}{c} E({\hat{H}}_{l})\geq -\sum_{i=1}^{4}p_{i}\;\text{log}(\frac{(N-1)p_{i}+1}{N})\\ E({\hat{H}}_{l})\leq \sum_{i=1}^{4}-p_{i}\;\text{log}(\frac{p_{i}}{1-{\left(1-p_{i}\right)}^{N}}) \end{array}$$

A full derivation of this relation is given in the Appendix. We note that the upper limit for the measured sequence entropy is strictly less than the true sequence entropy in Equation (1); this result implies that a measurement of entropy from sequence data is likely to underestimate the true entropy of the population. We further note that, as the read depth *N* increases, both the lower and upper bounds in our formula increase, both tending to the correct value. This implies that the expected shortfall in the entropy given by the calculation will depend upon the read depth of sequencing. As a consequence entropy, when calculated in this way, is not a good measure of sequence diversity. If two different populations are sequenced to different read depths, values of the entropy calculated for the two populations may or may not reflect the ordering of the true levels of population diversity.

**Figure 1.**
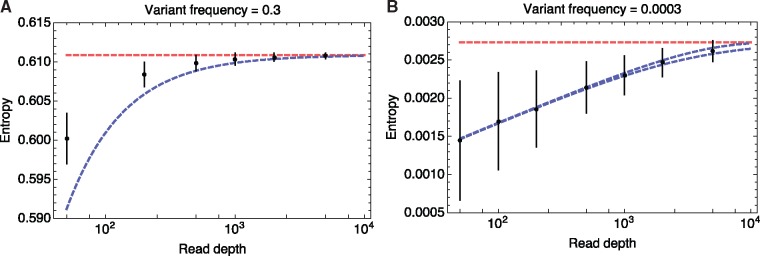
Mean sequence entropy values calculated for sets of 1,000 loci each of which has a consistent minor variant frequency. Means of these values calculated across 100 replicates are shown as black dots, with vertical bars, where visible, showing an interval of ±2 standard deviations. The correct entropy is shown by a dashed red line. The dashed blue lines, where not obscured by the correct entropy value, show the upper and lower limits described in Equation (3), with the upper limit showing the correct sequence entropy value. Data are shown for (A) a variant frequency of 30% and (B) a variant frequency of 0.03%.

To investigate the effect of read depth upon the calculated sequence entropy, we performed calculations for simulated data describing high- and low-frequency polymorphisms. For a variant at intermediate frequency, namely 30% of the population, the mean calculated sequence entropy falls between the two limits of Equation (3), increasing with increasing read depth (Fig. 1A). At read depths of 1,000 or less, there is a noticeable shortfall in the entropy with respect to the true sequence diversity. Variants at lower frequencies lead to more incorrect entropy values at higher depths of sequencing; as shown in Fig. 1B, the lower bound remains below the true value for much longer. In so far as viral populations contain large numbers of low-frequency variants, our result implies that a depth-dependent shortfall in the measurement of entropy will be pervasive even at high read depths. The measure of entropy obtained will depend upon the extent to which a population has been sequenced.

### 3.2 Number of polymorphisms per kilobase

The number of polymorphisms per kilobase of genome is calculated relative to a definition of what constitutes a polymorphism, usually a minor allele frequency between 1% and 5% (Xue et al. 2017; McCrone et al. 2018); given this definition, calculation of the statistic is trivial. This statistic has been used to compare the diversity of reported influenza populations, highlighting potential discrepancies in the genome sequencing of some datasets (Xue and Bloom 2018). An alternative measure of viral diversity, the ‘richness’ of a viral population, is calculated as the total number of polymorphisms in the viral genome (McCrone and Lauring 2016); the two statistics are straightforwardly related.

**Figure 2.**
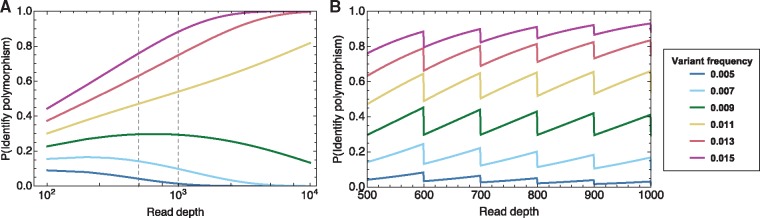
(A) Trend in the probability of a variant being identified as a polymorphism at 1% frequency as a function of read depth. At very high read depth, variants with a frequency greater than 1% will always be identified as polymorphisms, while variants below this frequency will never be identified as polymorphisms. Details of the function in the region between the vertical grey dashed lines are shown in (B). Detailed probability values. The range of frequencies at which a variant can be identified is constrained to the set of values *i*/*N* where *N* is the read depth; this constraint leads to a sawtooth pattern in the probability of identifying a polymorphism.

Calculations show that the measured number of polymorphisms per kilobase is also dependent upon read depth, albeit that the influence of read depth is more complex than is the case for sequence entropy. To illustrate this, we suppose that a threshold frequency of 1% is used to define the existence of a polymorphism. Given a binomial sample of depth *N*, an allele will be identified as polymorphic if at least *n* copies of the minority allele are observed, where n/N≥0.01. If the true variant frequency is given by *p*, the probability of identifying a polymorphism is given by the cumulative distribution function:

(4) $$P\left(\frac{n}{N}\geq 0.01\right)=\sum_{i=k}^{N}\frac{N!}{i!(N-i)!}p^{i}{\left(1-p\right)}^{N-i}$$

where *k* is the minimum value for which k/N>0.01; the broad-scale behaviour of this function is shown in Fig. 2. While this function is non-monotonic in *N*, it is straightforward to observe that, as *N* becomes large, the probability of identifying a polymorphism tends towards 0 if *P *<* *0.01, tends towards 1 if *P *>* *0.01, and tends towards 0.5 if *P *=* *0.01. (The probability is further influenced by discrete-value effects, illustrated in Fig. 2B.)

In so far as the chances of identifying a single polymorphism are influenced by the read depth, the expected number of polymorphisms per thousand bases is also dependent upon *N*. In a system for which a large number of variants are polymorphic at frequencies less than, but close to 1%, the number of identified polymorphisms will decrease at higher read depths, as higher precision observations show these variants to be below the polymorphism threshold. Conversely, if a large number of loci are polymorphic at frequencies slightly above 1%, an increase in read depth will cause the expected diversity also to increase. Since changes in sequencing depth can both increase and decrease the number of polymorphisms identified, this statistic is not so affected by read depth as the calculation of entropy. However, it is not an ideal statistic for the comparison of samples; statistics calculated for samples with different read depth profiles may not be formally comparable.

### 3.3 Nucleotide diversity *π*

The diversity statistic *π* was first derived for the comparison of sequences in a phylogenetic tree (Nei and Li 1979), but can be applied to viral sequence data even where full genomes are not available (Nelson and Hughes 2015). As with other measures, this statistic has been applied to evaluate both to compare diversity values, and to evaluate changes in diversity in viral populations over time (Maldarelli et al. 2013; Dinis et al. 2016). At the locus *l*, where *n_i_* copies of the allele *i* are observed, the proportion of pairwise differences between alleles may be calculated as

(5) $$D_{l}=\frac{\sum_{i\neq j}n_{i}n_{j}}{\frac{1}{2}N(N-1)}=\frac{N(N-1)-\sum_{i}n_{i}(n_{i}-1)}{N(N-1)}$$

The statistic *π* may then be calculated for a genome as

(6) $$\pi =\sum_{l=1}^{L}D_{l}/L$$

Calculating an expression for the expected value of *D_l_* showed it not to be dependent upon the depth of sequencing, but only upon the underlying frequencies of the alleles at this locus.

(7) $$E(D_{l})=1-\sum_{i=1}^{4}p_{i}^{2}$$

Derivation of this result is shown in the Appendix. Here we see that, unlike the statistics considered above, this value does not depend upon the depth at which the locus is sampled, being a function only of the underlying allele frequencies *p_i_*. As such, where samples with different read depths are compared, the statistic *π* should not cause systematic biases in the reported results. We note that the variance of the statistic *D_l_* is dependent upon *N*: higher read depths are likely to generate more precise estimates of diversity.

### 3.4 Application to viral sequence data

**Figure 3.**
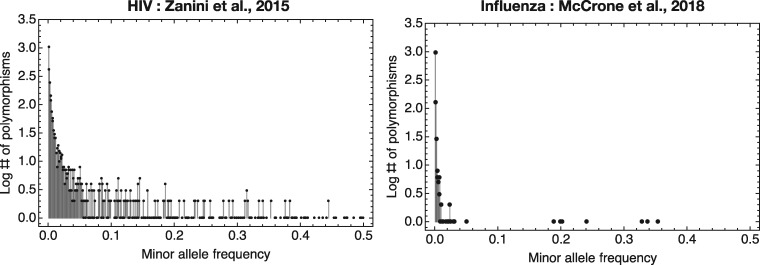
Allele frequency spectra for the two datasets analysed in this study. The within-host influenza dataset shows a small number of polymorphic sites relative to the HIV data.

In order to investigate the effect of depth-dependent biases upon diversity statistics when applied to biological sequence data, we analysed published data describing within-host HIV and influenza populations (Zanini et al. 2015; McCrone et al. 2018). Data were chosen to represent contrasting viral populations when evaluated in terms of sequence diversity; plots of allele frequency spectra for each dataset are shown in Fig. 3.

**Figure 4.**
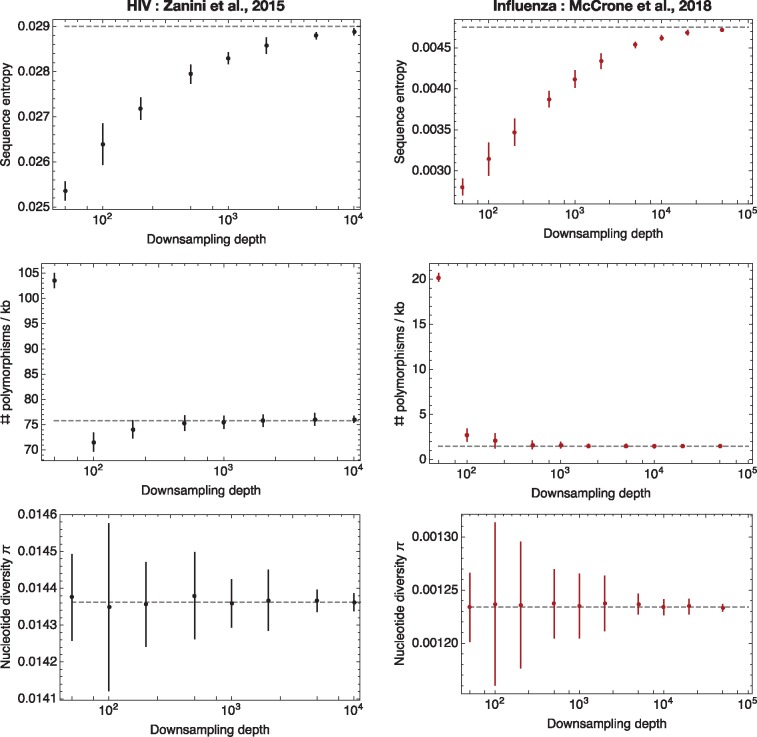
Diversity statistics calculated for HIV (black) and influenza (red) sequence data following downsampling of the data to lower read depths. Ten replicate downsampling calculations were performed for each point; dots show mean values, with vertical bars, where visible, showing an interval of ±2 standard deviations. Dashed grey lines show the values calculated from the complete dataset.

Downsampling of data from each population showed substantial changes in the calculated sequence entropy as the number of reads was altered (Fig. 4). For example, downsampling the influenza dataset to a depth of 100 led to a calculated entropy value only 66.1% of the ‘correct’ value, calculated from the original data. Even where data were downsampled to a read depth of $5\times 10^{4}$, the calculated entropy was still fractionally lower than the value calculated for the dataset as a whole.

With the exception of values measured at the lowest downsampling depths, calculations of the number of polymorphisms per kb showed relatively smaller changes with read depth. We note that in the HIV dataset, after an initial fall, this statistic increased with read depth, while in the influenza dataset the statistic gradually decreased to the correct value; the precise distribution of frequencies affects the manner in which this statistic is biased by sample depth.

Calculations of the nucleotide diversity *π* showed roughly constant results across downsampled datasets. No clear relationship between this statistic and the downsampling depth was observed; the diversity calculated from the complete dataset was always encompassed in the range of values obtained from downsampled data.

## 4. Discussion

Viral sequence diversity is an important property in the evolution of viral populations. While diversity is complex, statistics which measure it as a single numerical value provide a useful tool for the comparison of viral datasets, either across genome sequencing studies, or within the course of a single infection. Here, assessing three commonly used such statistics, we have highlighted potentially severe problems in the use of sequence entropy, with lesser though potentially important issues with the number of polymorphisms per kb of genome. Issues arise with these statistics due to the inherent dependence of each upon the read depth of sampling. Entropy is dependent upon read depth in a systematic way, with greater sampling giving a higher estimate of diversity. The number of polymorphisms per kb is dependent upon depth in a more complex manner; greater sampling may increase or decrease the value of this metric.

The depth-dependence of statistics shown here matters in cases where such statistics are used to compare diversity between different populations. Differences either in the overall read depth, or in the distribution of read depth across the genome, could produce misleading results if poor quality statistics are used for the evaluation of population-level diversity. While technologies such as the Illumina HiSeq can be used to achieve very high read depths, the use of appropriate statistics is a more efficient approach for the evaluation of sequence diversity. We note that diversity statistics may also be applied to evaluate data at the between-host level (Renzette et al. 2017). Such analyses may involve lower sequence ‘depths’ than within-host data. Care in the analysis of both within- and between-host diversity measurements is required.

We here make two recommendations. Firstly, where a variety of statistics have been used to measure viral diversity, we propose that the nucleotide diversity *π* outperforms other metrics in providing an estimator that is unbiased by factors of genome sequencing. Particularly where samples with different read depth profiles are compared, this metric allows the fair evaluation and comparison of sequence diversity. While corrections allowing the unbiased estimation of entropy can be made (Montgomery-Smith and Schürmann 2014), the simplicity and general acceptance of *π* by the evolutionary community make this, in our opinion, the favoured solution. Secondly, we propose that where diversity statistics are compared, an estimate of the uncertainty of such statistics should also be made. In being generated from genome sequence data, which describes the output of a random sampling process, diversity statistics are themselves statistical entities. Processes such as bootstrapping, the resampling of datasets from the allele frequencies they originally report, can give a straightforward estimate of the uncertainty in a given diversity measurement.

## 1. Mathematical appendix

We here derive the results stated in the main text. In our calculations, we represent the process of sequencing as one of sampling with replacement, giving rise to a multinomial formulation; this assumes the within-host viral population to be small. We note that the alternate assumption, of sampling without replacement, leads to similar results.

### 1.1 Expected value of sequence entropy

The expected entropy, described in Equation (3) of the main text, assuming multinomial sampling, can be written as follows:

(A.1) $$E(H_{l})=\sum_{\left\{n_{k}|\sum n_{k}=N\right\}}\left[\left(\sum_{i=1}^{4}-\frac{n_{i}}{N}\text{log}\;\left(\frac{n_{i}}{N}\right)\right)\left(\frac{N!}{\prod_{i}n_{i}!}\prod_{i}{\left(p_{i}\right)}^{n_{i}}\right)\right]$$

Rearranging this equation, we obtain

(A.2) $$E(H_{l})=\sum_{i=1}^{4}p_{i}\sum_{\left\{n_{k}|\sum n_{k}=N,n_{i}\geq 1\right\}}\;\text{log}\;\left(\frac{N}{n_{i}}\right)(N-1)!\frac{{\left(p_{i}\right)}^{n_{i}-1}}{(n_{i}-1)!}\prod_{j\neq i}\frac{{\left(p_{j}\right)}^{n_{j}}}{n_{j}!}$$

Next, applying Jensen’s inequality, we obtain

(A.3) $$\begin{array}{cc} E(H_{l}) & \leq \sum_{i=1}^{4}p_{i}\;\text{log}\;\left[\sum_{\left\{n_{k}|\sum n_{k}=N,n_{i}\geq 1\right\}}\left(\frac{N}{n_{i}}\times (N-1)!\frac{{\left(p_{i}\right)}^{n_{i}-1}}{(n_{i}-1)!}\prod_{j\neq i}\frac{{\left(p_{j}\right)}^{n_{j}}}{n_{j}!}\right)\right]\\ & =\sum_{i=1}^{4}p_{i}\;\text{log}\;\left[\frac{1}{p_{i}}\sum_{\left\{n_{k}|\sum n_{k}=N,n_{i}\geq 1\right\}}N!\frac{{\left(p_{i}\right)}^{n_{i}}}{n_{i}!}\prod_{j\neq i}\frac{{\left(p_{j}\right)}^{n_{j}}}{n_{j}!}\right]\\ & =\sum_{i=1}^{4}-p_{i}\;\text{log}\;\left[\frac{p_{i}}{1-{\left(1-p_{i}\right)}^{N}}\right]\\ & <\sum_{i=1}^{4}-p_{i}\;\text{log}\;\left(p_{i}\right) \end{array}$$

To get the lower bound, we again apply Jensen’s inequality to Equation (A.2).

(A.4) $$\begin{array}{cc} E(H_{l}) & \geq -\sum_{i=1}^{4}p_{i}\;\text{log}\;\left[\sum_{\left\{n_{k}|\sum n_{k}=N\right\}}\left(\frac{n_{i}}{N}\times (N-1)!\frac{p_{i}^{n_{i}-1}}{(n_{i}-1)!}\prod_{j\neq i}\frac{p_{j}^{n_{j}}}{n_{j}!}\right)\right]\\ & =-\sum_{i=1}^{4}p_{i}\;\text{log}\;\left[\frac{1}{N}+\frac{(N-1)p_{i}}{N}\sum_{\left\{n_{k}|\sum n_{k}=N,n_{i}\geq 2\right\}}\left((N-2)!\frac{p_{i}^{n_{i}-2}}{(n_{i}-2)!}\prod_{j\neq i}\frac{p_{j}^{n_{j}}}{n_{j}!}\right)\right]\\ & =\sum_{i=1}^{4}-p_{i}\;\text{log}\;\left[\frac{(N-1)p_{i}+1}{N}\right] \end{array}$$

Examining the two bounds, we note that

(A.5) $$\underset{N\to \infty }{\lim}\sum_{i=1}^{4}-p_{i}\;\text{log}\;\left[\frac{(N-1)p_{i}+1}{N}\right]=\sum_{i=1}^{4}-p_{i}\;\text{log}(p_{i})$$

and

(A.6) $$\underset{N\to \infty }{\lim}\sum_{i=1}^{4}-p_{i}\;\text{log}\;\left(\frac{p_{i}}{1-{\left(1-p_{i}\right)}^{N}}\right)=\sum_{i=1}^{4}-p_{i}\;\text{log}(p_{i}).$$

We therefore have the result

(A.7) $$\underset{N\to \infty }{\lim}E(H_{l})=\sum_{i=1}^{4}-p_{i}\;\text{log}\;\left(p_{i}\right).$$

### 1.2 Expected value of π and its variance

The expected value of the statistic *D_l_* in Equation (5) for a specific locus *l* can be expressed as follows:

(A.8) $$\begin{array}{ccc} E\left\{D_{l}\right\} & = & \sum_{\left\{n_{k}|\sum n_{k}=N\right\}}\frac{N(N-1)-\sum_{i=1}^{4}n_{i}(n_{i}-1)}{N(N-1)}N!\prod_{j=1}^{4}\frac{{\left(p_{j}\right)}^{n_{j}}}{n_{j}!}\\ & = & 1-\frac{1}{N(N-1)}\sum_{i=1}^{4}\sum_{\left\{n_{k}|\sum n_{k}=N\right\}}n_{i}(n_{i}-1)N!\prod_{j=1}^{4}\frac{{\left(p_{j}\right)}^{n_{j}}}{n_{j}!}\\ & = & 1-\sum_{i=1}^{4}p_{i}^{2}\sum_{\left\{n_{k}|\sum n_{k}=N\right\}}\left(N-2\right)!\frac{{\left(p_{i}\right)}^{n_{i}-2}}{(n_{i}-2)!}\prod_{j\neq i}\frac{{\left(p_{j}\right)}^{n_{j}}}{n_{j}!}\\ & = & 1-\sum_{i=1}^{4}p_{i}^{2} \end{array}$$

Here the value $1-\sum_{i=1}^{4}p_{i}^{2}$ is the true proportion of pairwise differences for the locus *l*; our result is independent of *N*. Hence, the statistic *D_l_* and the linear combination of these values, *π*, are unbiased with respect to the depth of sequencing.

We note that the variance of *D_l_* can also be expressed as the function of *p_i_* and *N*,

(A.9) $$\begin{array}{ccc} \text{Var}\left\{D_{l}\right\} & = & \sum_{\left\{n_{k}|\sum n_{k}=N\right\}}\frac{{\left[\sum_{i=1}^{4}n_{i}(n_{i}-1)\right]}^{2}}{N^{2}{\left(N-1\right)}^{2}}N!\prod_{j=1}^{4}\frac{{\left(p_{j}\right)}^{n_{j}}}{n_{j}!}-{\left[\sum_{i=1}^{4}p_{i}^{2}\right]}^{2}\\ & = & \frac{2}{N(N-1)}\sum_{i}p_{i}^{2}\left[1+(2N-4)p_{i}-(2N-3)\sum_{j}p_{j}^{2}\right]\\ & = & \frac{2}{N(N-1)}\sum_{i}p_{i}^{2}\left[1-\sum_{i}p_{i}^{2}\right]+\frac{4(N-2)}{N(N-1)}\left[\sum_{i}p_{i}^{3}-{\left(\sum_{i}p_{i}^{2}\right)}^{2}\right]. \end{array}$$

Generalising this result, the variance of *π* may therefore be said to be proportional to 1/*N*. It would be expected to tend to zero as the read depth becomes large.
